# Self-Reported Quality of Life and 30-Day Mortality in Elective Cardiac Surgery

**DOI:** 10.1016/j.jacadv.2026.102768

**Published:** 2026-05-27

**Authors:** Lieke van Susante, Yuri Ganushchak, Elham Bidar, Peyman Sardari Nia

**Affiliations:** a Department of Cardiothoracic Surgery, Heart and Vascular Center, Maastricht University Medical Center, Maastricht, the Netherlands; b Department of Cardiothoracic Surgery, Cardiovascular Research Institute Maastricht, (CARIM), Maastricht University, Maastricht, the Netherlands

**Keywords:** cardiac surgery, mortality, patients, quality of life

## Abstract

**Background:**

Health-related quality of life (HRQoL) is increasingly assessed in cardiac surgery research, yet its association with hard clinical endpoints, such as mortality, remains underexplored. Demonstrating such an association may support HRQoL use in preoperative risk assessment.

**Objectives:**

The purpose of this study was to evaluate the association between preoperative self-reported HRQoL (Short-Form Health Survey) and 30-day mortality in patients undergoing elective cardiac surgery.

**Methods:**

This retrospective cohort study analyzed prospectively collected data from adults undergoing cardiac surgery at Maastricht University Medical Center (2018-2023). Preoperative Short-Form Health Survey T-scores were used in a 2-step cluster analysis to identify higher and lower HRQoL groups. Thirty-day mortality and composite morbidity were compared between clusters. Physical (Physical Component Summary) and mental (Mental Component Summary) scores were analyzed in multivariable Cox models, with effects expressed per 10-point increase.

**Results:**

Among 2,259 patients, 55 (2.43%) died within 30 days. Mortality was lower in the higher HRQoL group (1.38% vs 3.71%; *P* < 0.001). Lower HRQoL was independently associated with higher mortality (adjusted HR: 2.22; 95% CI: 1.23-4.02; *P* = 0.008). Composite morbidity occurred less often in the higher HRQoL group (8.5% vs 11.4%; P = 0.026). Among patients with complications, mortality was lower in the higher HRQoL group (11.8% vs 23.0%; *P* = 0.031). Higher Physical Component Summary, but not Mental Component Summary, was associated with lower mortality (HR per 10-point increase 0.66; 95% CI: 0.49-0.91; *P* = 0.01).

**Conclusions:**

Lower preoperative HRQoL was independently associated with higher 30-day mortality following elective cardiac surgery. Increased mortality following complications in patients with lower HRQoL suggests limited physiological reserve. Incorporating HRQoL, particularly physical health, may improve preoperative risk stratification.

Traditionally, the effectiveness of treatments in cardiovascular interventions has been evaluated primarily through clinical outcomes such as mortality and morbidity. However, over the past decade, there has been an increasing interest in patient-measured and patient-experienced outcomes, particularly health-related quality of life (HRQoL).^1^, ^2^, ^3^ This shift in interest is not only reflected in research but also in clinical practice, where preoperative HRQoL assessments are increasingly incorporated into decision-making to estimate the potential benefits of an intervention for an individual patient.^4^ HRQoL assessments provide insights into the impact of both the disease and its treatment on the patient’s overall well-being, offering a more comprehensive evaluation of treatment effectiveness beyond traditional clinical measurements.^5^^,^^6^

However, despite the recognition of patient-reported outcomes in evidence-based medicine, the QoL remains underused.^7^^,^^8^ HRQoL may have relevance beyond its traditional role as a secondary endpoint, as it may provide independent prognostic information that exceeds the traditional risk score. HRQoL integrates multiple domains, such as frailty, psychosocial functioning, and physiological reserve, that are only partially represented in established surgical risk scores. By integrating these broader patient-reported domains, HRQoL may improve the prediction of adverse outcomes, including mortality, thereby offering additional clinical value and possibly surpassing other surgical risk scores.

In the contemporary clinical trials, there is no established association made between the QoL and clinical outcomes such as mortality. Recognizing the value of QoL as a potential factor associated with survival duration could enhance the practical application of QoL assessments, facilitating more accurate preoperative risk stratification and improve individualized decision-making.^5^

Therefore, the aim of this study was to evaluate the possible association between preoperative self-reported HRQoL, using the Short-Form Health Survey (SF-36v2) survey, and 30-day mortality in patients undergoing elective cardiac surgery.

## Material and methods

### Study design

A retrospective cohort study was conducted investigating the association between preoperative HRQoL, as assessed by the SF-36v2 survey at baseline before surgery, and 30 day mortality in patients undergoing elective cardiac surgery. All data were prospectively gathered and later audited and validated by the Netherlands Heart Registration. After this, the data were retrospectively extracted for this study. Our local ethical committee waived the need for informed consent due to the observational and retrospective nature of the study (METC 15–4-065) for first cohort publication and in the framework of project of development of DT (METC 2022-3135). The authors assume responsibility for the accuracy and completeness of the data and analyses. The data set analyzed during the current study is available from the corresponding author upon reasonable request.

### Patients

All patients who underwent elective cardiac surgery at Maastricht University Medical Center (MUMC+) in the Netherlands, in a period from January 2018 till end of 2023 were prospectively asked to complete a SF36v2 survey. Cardiac surgery was defined as coronary artery bypass grafting (CABG), valve surgery, aortic surgery, atrial fibrillation ablation, or other procedures (for combinations, see Table 1). The baseline questionnaire was sent via email prior to surgery. If the patients had not completed the questionnaire before their admission, they were asked by a physician during their physical preoperative consult to complete it on a provided tablet in the waiting area. Patients who did not complete the SF-36v2 survey, or completed it only partially, were excluded.

**Table 1:** Baseline Characteristics

Baseline Characteristics	Overall (N = 2,259)	Cluster 1 (n = 1,235)	Cluster 2 (n = 1,024)	*P* Value
Age, y	68.3 ± 10.4	68.7 ± 10.4	67.7 ± 10.4	0.027
Male (sex)	1,614 (71.4)	938 (76.0)	676 (66.0)	<0.001
BMI, kg/m^2^	27.6 ± 4.5	26.8 ± 4.1	28.4 ± 4.8	<0.001
EuroSCORE II	1.44 [0.94- 2.59]	1.39 [0.91-2.44]	1.54 [0.97-2.81]	<0.001
Diabetes mellitus	449 (19.9)	193 (15.6)	256 (25.0)	<0.001
LVEF	53.2 ± 8.3	53.3 ± 8.2	53.1 ± 8.3	0.463
eGFR, mL/min/1.73 m^2^	94.8 ± 45.7	91.6 ± 27.0	98.7 ± 60.9	<0.001
Chronic lung disease	189 (8.4)	76 (6.2)	113 (11.0)	<0.001
Previous heart surgery	220 (9.7)	94 (7.6)	126 (12.3)	<0.001
Critical preoperative state	20 (0.9)	5 (0.4)	15 (1.5)	0.007
Recent myocardial infarction	90 (4.0)	41 (3.4)	49 (4.8)	0.314
Extracardiac arteriopathy	203 (9.0)	88 (7.1)	115 (11.2)	<0.001
Poor mobility	97 (4.3)	22 (1.8)	75 (7.3)	<0.001
Active endocarditis	3 (0.1)	1 (0.1)	2 (0.2)	0.458
Pulmonary artery pressure (mm Hg)	27.1 ± 6.5	27.2 ± 6.3	27.3 ± 6.7	0.764
CCS class				
IV	42 (2.3) 1858/2,259	15 (1.5) 1,007/1,235	27 (3.2) 851/1,024	0.015
NYHA functional class				<0.001
I	620 (33.9) 1827/2,259	435 (43.9) 991/1,235	185 (22.1) 836/1,024	
II	779 (42.6) 1827/2,259	422 (42.6) 991/1,235	357 (42.7) 836/1,024	
III	387 (21.2) 1827/2,259	124 (12.5) 991/1,235	263 (31.5) 836/1,024	
IV	41 (2.2) 1827/2,259	10 (1.0) 991/1,235	31 (3.7) 836/1,024	
Surgery characteristics				<0.001
CABG	531 (23.5)	280 (22.7)	251 (24.5)	
MIDCAB	226 (10.0)	117 (9.5)	109 (10.6)	
OPCAB	39 (1.7)	16 (1.3)	23 (2.2)	
Aortic valve surgery	806 (35.7)	422 (34.2)	384 (37.5)	
Mitral valve surgery	173 (7.7)	115 (9.3)	58 (5.7)	
Tricuspid valve surgery	4 (0.2)	2 (0.2)	2 (0.2)	
Multiple valve combined surgery	66 (2.9)	29 (2.3)	37 (3.6)	
CABG + valve surgery	143 (6.3)	92 (7.4)	51 (5.0)	
CABG + aorta surgery	3 (0.1)	2 (0.2)	1 (0.1)	
CABG + aorta + valve surgery	14 (0.6)	11 (0.9)	3 (0.3)	
Aorta surgery	33 (1.5)	22 (1.8)	11 (1.1)	
Aorta surgery + valve surgery	95 (4.2)	63 (5.1)	32 (3.1)	
AF ablation	65 (2.9)	36 (2.9)	29 (2.8)	
Other	61 (2.7)	28 (2.3)	33 (3.2)	

Values are mean ± SD, median [IQR], or n (%).

AF = atrial fibrillation; BMI = body mass index; CABG = coronary artery bypass grafting; CCS = Canadian Cardiovascular Society; Cluster 1 = patients where health domain scores were higher than the “normal” range for our population, Cluster 2 = where health domain scores were lower than the “normal” range for our population, eGFR = estimated glomerular filtration rate; EuroSCORE II = European System for Cardiac Operative Risk Evaluation II; LVEF = left ventricular ejection fraction; MIDCAB = minimally invasive direct coronary artery bypass; OPCAB = off-pump coronary bypass.

### Measures

All baseline characteristics and the European System of Cardiac Operative Risk Evaluation (EuroSCORE II) of the patients were obtained preoperatively. Sex was defined as a biological variable (male/female) obtained from the medical record. Gender identity was not assessed.

The SF-36v2 consists of 36 questions used to measure 8 domains of health: 1) physical functioning; 2) role limitations due to physical health problems; 3) bodily pain; 4) general health; 5) vitality (energy/fatigue); 6) social functioning; 7) role limitations due to emotional problems; and 8) mental health (Scoring procedure algorithm see Supplemental methods). Each item is scored on a 0 to 100 scale, with higher scores indicating more favorable self-reported functioning or well-being within the respective domain. Domain scores are normalized to a mean of 50 ± 10 based on the general population, where 50 represents the average and each 10 points corresponds to one SD. Accordingly, a higher score in one domain (eg, physical functioning) does not preclude impairment in another (eg, mental health), reflecting the multidimensional nature of HRQoL.^8^

The endpoint was all-cause 30-day mortality after surgery. Survival data from January 2018 to December 2023 were included as an outcome parameter of the study. The survival rates were traced during the 30-day postoperative period by the Netherlands Heart Registration of the Netherlands based on the National Register of Deceased Persons. The secondary outcome, the composite morbidity (CM) rate within 30 days after surgery, was retrospectively obtained from hospital records. The composite outcome (CM) was defined as the occurrence of any major postoperative complication (including stroke, acute renal failure, deep sternal wound infection, reoperation, and prolonged ventilation) within 30 days after surgery.^9^

### Statistical analysis

The sociodemographic and clinical characteristics were analyzed using descriptive statistics, with percentages for categorical variables and either mean ± SD or median [IQR] for continuous variables depending on the symmetry of the distribution, with skewed data presented as median [IQR] and approximately symmetric data as mean ± SD. Baseline differences between the participants and nonparticipants and between the 2 clusters were analyzed using an independent *t*-test, chi-square test, or Mann-Whitney *U* test, as appropriate. The responses to the SF36v2 survey were scored using the algorithm described in the User's Manual for the SF-36v2 Health Survey (3rd ed)^10^ (See Supplemental Appendix A).

In the first step of the analysis, health domain T-scores for survivors and nonsurvivors were selected to identify a set of variables that differ in survivors and nonsurvivors. A one-way analysis of variance was performed for each domain. Parameters that showed statistically significant differences between the survivor and nonsurvivor (Supplemental Table 1) were selected for the clustering procedure. This preselection was performed to reduce dimensionality and to focus the clustering procedure on HRQoL domains most strongly related to the outcome.

In the second step, an unsupervised 2-step cluster analysis was performed using the significantly different health domain T-scores from the preoperative survey. The 2-step clustering algorithm implemented in SPSS (version 11.5 and later) is designed for large data sets and can accommodate both continuous and categorical variables. The method applies a 2-stage approach consisting of an initial preclustering procedure followed by hierarchical clustering. The optimal number of clusters is automatically determined by the algorithm based on model-fit criteria.

Cluster quality was evaluated using the average silhouette measure of cohesion and separation. This procedure resulted in 2 clusters: Cluster 1, where health domain scores were higher than the “normal” range for our population, and Cluster 2, where health domain scores were lower than the “normal” range.

The observed 30-day mortality in each cluster was calculated and compared with the corresponding EuroSCORE II values for each group. The EuroSCORE II was used to estimate the expected mortality. A Kaplan-Meier analysis was used to assess the unadjusted estimated survival rate in the 2 clusters. The association between HRQoL and mortality was further evaluated using a Cox proportional hazards regression analysis. Covariates were specified based on clinical relevance and baseline differences (*P* < 0.10). Results were presented as HRs with 95% CIs.

To investigate the potential effect of higher preoperative QoL on postoperative resilience, we examined the association between CM and 30-day postoperative mortality in both patient clusters. Categorical variables were compared using the Pearson chi-square test of independence. Separate chi-squared tests were performed within each cluster to assess whether the presence of CM was significantly associated with 30-day mortality.

To further explore the potential clinical relevance of preoperative HRQoL, we performed additional analyses evaluating preoperative HRQoL using the validated SF-36 summary measures: Physical Component Summary (PCS) and Mental Component Summary (MCS). These measures were included as continuous variables of 30-day mortality in multivariable Cox proportional hazards models, adjusted for age, sex, and EuroSCORE II. To explore potential nonlinear associations, we fitted restricted cubic splines with 4 knots. HRs per 10-point increase were estimated for interpretability. The proportional hazards assumption was assessed using Schoenfeld residuals.

Tests were considered statistically significant at the 95% CI (*P* < 0.05). Statistical analyses were performed using SPSS version 27 (SPSS Inc) and Rstudio.

## Results

### Baseline characteristics

A total of 2,593 patients underwent surgery during the inclusion period, of whom 2,259 patients were included in the study because they completed the preoperative SF-36 questionnaire. A total of 334 patients were excluded. Among the excluded patients, 260 (77.8%) were male, with a mean age of 66.7 ± 9.4 years, a mean body mass index (BMI) of 27.3 ± 4.1, and a median EuroSCORE II of 1.82 [1.09-2.92]. The most common procedures among excluded patients were CABG (n = 126; 37.7%), minimally invasive direct coronary artery bypass (n = 42; 12.6%), and aortic valve surgery (n = 33; 9.9%). Of the 2,259 patients who completed the questionnaire before surgery, 1,614 (71.4%) were male, the mean age was 68.3 ± 10.4, and 449 (19.9%) had diabetes mellitus. The mean BMI was 27.6 ± 4.5 and the median EuroSCORE was 1.44 [0.94- 2.59].

An overview of all baseline characteristics and procedures is presented in Table 1. The most performed procedures were aortic valve surgery, CABG, and minimally invasive direct coronary artery bypass.

The Canadian Cardiovascular Society class was missing for 401 patients. Among those 1,858 patients, 42 (2.3%) were categorized in the Canadian Cardiovascular Society class IV. The NYHA functional class was missing for 432 patients. Of the 1,827 patients, 620 (33.9%) were categorized in NYHA functional class 1, 779 (42.6%) in class II, 387 (21.1%) in class III, and 41 (2.2%) in class IV.

Non participants were significantly younger, more often male, and had a higher EuroSCORE II compared with participants. Participants more frequently underwent aortic valve surgery.

In the comparison between clusters, patients in Cluster 1 were significantly older, more often male, had a lower BMI, and had a lower EuroSCORE II score (Table 1).

### Association between 30-day mortality and the health domain scores

To investigate which set of variables of the health domain T-scores are different in survivors and nonsurvivors, a one-way analysis of variance was done. The mean health domain T-scores for survivors and nonsurvivors are presented in the Supplemental Appendix B. The mean health domain T-scores in survivors for each domain had a range of 50.0 to 50.6, with an SD range of 9.3 to 10.0. The mean health domain T-scores in nonsurvivors were between 45.3 and 50.0, with an SD range of 9.3 to 10.5.

Parameters that were significantly different between survivors and nonsurvivors were selected for the 2-step clustering procedure. These included general health, vitality, social functioning, and PCS. Despite statistically significant differences, physical functioning and role-physical were excluded from the analysis as they are integral components of the PCS.

After the 2-step clustering procedure, all patients (2,259) were divided in 2 clusters based on their physical and mental health status results on the SF36v2 survey. Cluster 1, where health domain scores were higher, included 1,235 patients (54.7%). Cluster 2, where health domain scores were lower, consisted of 1,024 patients (45.3%). The average silhouette measure of cohesion and separation was 0.5, indicating reasonable cluster separation.

The mean physical and mental health status of patients before surgery in each cluster is shown in Supplemental Figure 1. The T-scores for all health domains were significantly lower in Cluster 2 compared to Cluster 1 (*P* < 0.001).

**Figure 1:**
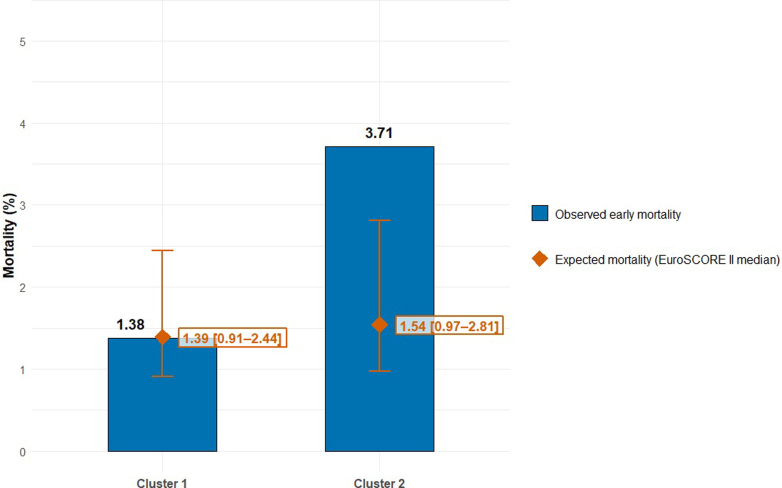
Observed vs Expected 30-Day Mortality by Cluster This figure compares observed and European System for Cardiac Operative Risk Evaluation II expected 30-day mortality across the 2 Short-Form Health Survey-derived clusters in the elective cardiac surgery cohort (N = 2,259). Observed mortality was 1.38% (17/1,233) in Cluster 1 and 3.71% (38/1,026) in Cluster 2 (*P* < 0.001). The observed mortality in Cluster 1 was lower than expected, whereas Cluster 2 exceeded its expected mortality. EuroSCORE II = European System for Cardiac Operative Risk Evaluation II.

Of the 2,259 patients, 55 (2.43%) patients died within 30 days after surgery. Of the 55 patients who died, 17 patients were categorized in Cluster 1 and 38 patients were included in Cluster 2. The 30-day mortality rate for patients in Cluster 1 was 1.38%, compared to 3.71% in Cluster 2 (*P* < 0.001) (Figure 1). The observed mortality in Cluster 1 was lower than the expected mortality, which was estimated using the median EuroSCORE II. In Cluster 2, the observed mortality was higher than expected mortality.

**Figure 2:**
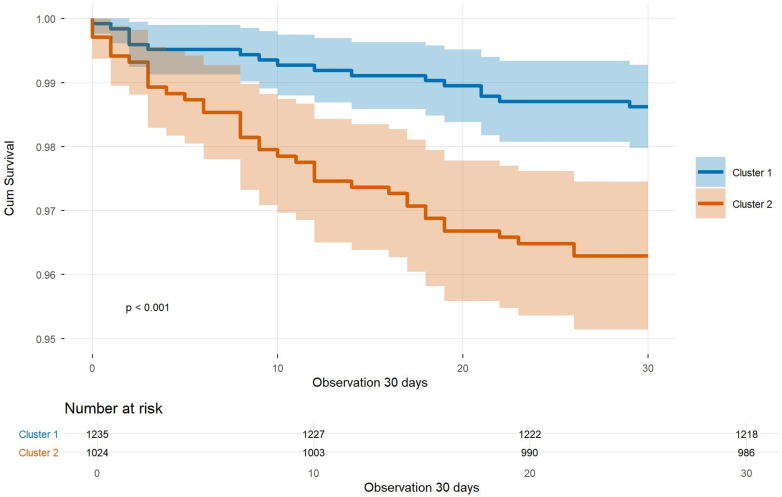
30-Day Survival by Cluster (Kaplan-Meier) Kaplan-Meier curves display 30-day postoperative survival for Cluster 1 and Cluster 2. Patients in Cluster 2 showed significantly lower survival throughout follow-up (log-rank *P* < 0.001), consistent with their higher early postoperative mortality. A table beneath the curves provides the number of patients at risk at each time point.

The unadjusted Kaplan-Meier survival analysis demonstrated a significantly lower estimated survival rate for patients in Cluster 2 log-rank test *P* < 0.001) (Figure 2).

The multivariable Cox proportional hazards regression analysis, which adjusted for EuroSCORE II and BMI, demonstrated that HRQoL was independently associated with 30-day mortality. Patients with lower preoperative HRQoL had a significantly higher risk of 30-day mortality compared to those with a higher HRQoL (HR: 2.22; 95% CI: 1.23-4.02; *P* = 0.008).

### The association between CM and 30-day postoperative mortality in both clusters

The overall frequency of CM differed between clusters, with a lower incidence in Cluster 1 compared to Cluster 2 (8.5% vs 11.4%). Among patients who developed postoperative complications, 30-day mortality differed between clusters. In Cluster 1, mortality was 11.8% (12 of 102), compared to 23.0% (26 of 113) in Cluster 2. This difference was statistically significant (chi-square [1, N = 215] = 4.658; *P* = 0.031).

### Associations between preoperative PCS and MCS scores and 30-day mortality

In multivariable Cox proportional hazards models, higher preoperative PCS scores were associated with a lower estimated risk of 30-day mortality (HR per 10-point increase: 0.66; 95% CI: 0.49-0.91; *P* = 0.01). Restricted cubic spline analysis indicated a largely linear relationship (*P* for nonlinearity = 0.43). MCS scores were not significantly associated with 30-day mortality (HR per 10-point increase: 1.11; 95% CI: 0.82-1.50; *P* = 0.49), and spline analysis showed no evidence of a nonlinear association (*P* for nonlinearity = 0.35). The proportional hazards assumption was satisfied (global test *P* = 0.10).

## Discussion

This study demonstrates that the preoperative patient-reported health status, as measured by SF-36v2 health domain scores, was independently associated with 30-day mortality in elective cardiac surgery patients. This association persisted after adjustment for EuroSCOREII and BMI, showing that patients with lower preoperative health profiles had more than a twofold increased hazard of 30-day mortality compared with those with a higher HRQoL. Although the incidence of postoperative complications differed modestly between clusters, mortality among patients with complications was significantly higher in the lower HRQoL group.

Complementary analyses showed that higher PCS scores, but not MCS scores, were associated with lower 30-day mortality, suggesting that physical health may be the primary driver of short-term postoperative survival. Overall, these findings suggest that preoperative HRQoL might have an additional clinical value to preoperative assessment, as it might captures patient frailty, psychosocial functioning, and physiological reserve, which is not fully reflected by conventional surgical risk scores (Central Illustration).

**Central Illustration:**
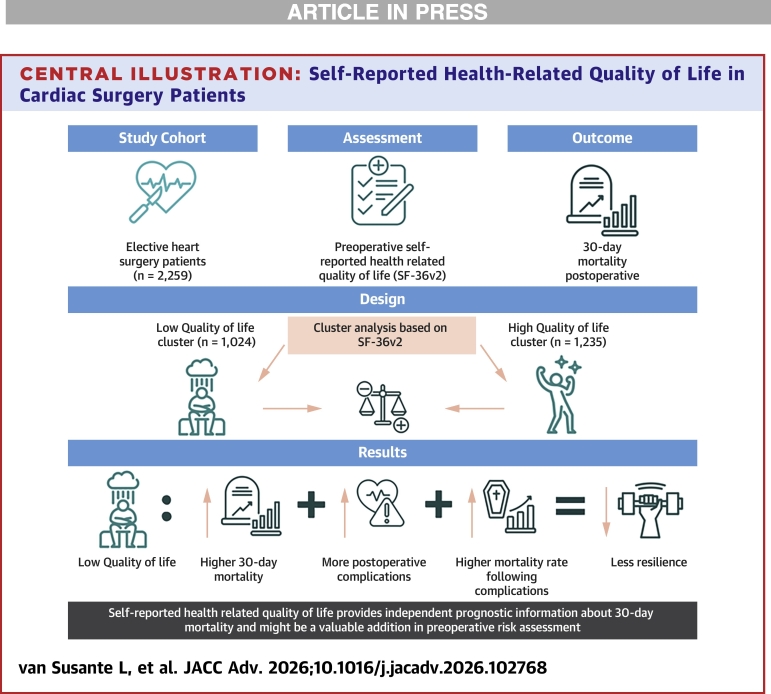
Self-Reported Health-Related Quality of Life in Cardiac Surgery Patients This central illustration summarizes a cohort of 2,259 adults undergoing elective cardiac surgery who completed a preoperative self-reported health status questionnaire (Short-Form Health Survey) and were monitored for 30-day postoperative mortality. A 2-step cluster analysis of Short-Form Health Survey scores identified a low health-related quality of life and a high health-related quality of life cluster, which were compared. The low health-related quality of life cluster exhibited higher 30-day mortality. The incidence of postoperative complications was modestly higher in the low health-related quality of life group. Among patients with complications, mortality was substantially higher in those with lower health-related quality of life, suggesting reduced physiological reserve and recovery capacity. Preoperative health-related quality of life provides independent prognostic information and may add value to risk assessment beyond traditional scores. SF-36v2 = Short-Form Health Survey. Symbols: Heart + scalpel icon: elective cardiac surgery patients. Questionnaire icon: Short-Form Health Survey self-reported preoperative quality of life. Gravestone with chart: 30-day postoperative mortality. Figure with dark cloud: low health-related quality of life. Figure with stars: high health-related quality of life. Heart + warning symbol: postoperative complications. Cluster block with 2 arrows: division into 2 health-related quality of life clusters. Upward arrow next to gravestone icon: increased 30-day mortality in low health-related quality of life cluster. Upward arrow next to complications symbol: more postoperative complications. Coffin with rising graph + upward arrow: higher mortality after complications in the low health-related quality of life cluster. ‘=’ symbol: potential explanatory mechanism. Arm with weight + downward arrow: reduced resilience in low health-related quality of life patients.

### Interpretation

The initial two-step cluster analysis algorithm based on the SF-36v2 survey defines two cluster in our study group. The cluster analysis methods used in this study are increasingly and successfully used in multiple clinical studies.^11^, ^12^, ^13^, ^14^ An possible explanation for the observed association between better preoperative quality of life and lower early postoperative mortality is that HRQoL reflects aspects of mental and physiological resilience that influence the ability to recover from complications. Patients with better preoperative QoL may have greater mental and physiological resilience, enabling them to recover more effectively from postoperative complications. Consistent with this, although differences in complication rates were modest, mortality among patients with complications was substantially higher in those with lower HRQoL. This suggests that postoperative outcomes are influenced not only by the occurrence of complications but also by the capacity to recover from them.

Our complementary analyses showed that preoperative PCS scores were significantly associated with 30-day mortality, with each 10-point increase in PCS linked to a 34% lower risk of 30-day mortality. This finding suggest that physical health, likely reflecting functional reserve and frailty, may be the primary driver of short-term postoperative survival. In contrast, MCS scores were not significantly associated with early mortality, indicating that mental health may have a more limited impact on immediate postoperative mortality, potentially influencing longer-term recovery instead. Importantly, the absence of a significant effect for MCS does not contradict the cluster-based findings. Clusters integrate multiple HRQoL domains, capturing subtle patterns of vulnerability that may not be apparent when examining single domains individually. Therefore, cluster-based differences in 30-day mortality likely reflect the combined contribution of physical and other HRQoL dimensions rather than mental health alone.

### Comparison with previous studies

Previous studies establish the effect of QoL on mortality in CABG surgery patients. Rumsfeld et al^5^ report a significant association between the PCS score and 6-month mortality, consistent with our findings. However, their study did not identify a relationship between the MCS score and mortality. A study by Curtis et al^15^ also find that a lower PCS score is associated with increased in-hospital mortality in CABG patients. However, in this study, they find that a decrease in mental health decreases the odds of mortality. Lastly, Masafumi Ono et al^16^ demonstrate that patients, who undergo CABG surgery, with the best physical and mental health have the best 10-year survival. These findings support our findings and the hypothesis that HRQoL plays a role in postoperative mortality. However, it should be noted that all previous studies focus on patients who undergo CABG procedures, in contrast to our population, which includes all elective cardiac surgery patients.

Our hypothesis that HRQoL reflects aspects of mental and physiological resilience that influence the ability to recover from complications is also in line with previous research, which found a strong association between better preoperative physical and mental health and lower postoperative mortality, likely due to greater tolerance of surgical stress and faster recovery capacity.^16^ This hypothesis is also supported by previous research demonstrating that high preoperative anxiety in cardiac surgery patients results in higher postoperative mortality and lower physical health is associated with mortality.^17^, ^18^, ^19^, ^20^ In addition, a study shows that patients who are physically and mentally better prepared for cardiac surgery experience fewer postoperative complications and a reduced mortality risk.^21^

### Clinical implications

The EuroSCORE II score is widely used to estimate operative mortality risk and guide decision-making regarding surgical risks.^22^^,^^23^ However, when comparing expected mortality (based on EuroSCORE II) with the observed mortality in our clusters, notable differences emerged. Patients in Cluster 1 had a lower observed mortality than estimated by EuroSCORE II, whereas patients in Cluster 2 had a higher observed mortality than expected.

One possible explanation for this difference is that aspects of HRQoL, particularly multidimensional impairments including frailty, psychosocial functioning, and physiological reserve, are not fully captured within the EuroSCORE II. As a result, EuroSCORE II may underestimate the postoperative resilience in patients with better HRQoL, resulting in a lower mortality rate. Incorporating preoperative HRQoL alongside EuroSCORE II in risk assessment may therefore improve the estimation of postoperative mortality risk.

Additionally, preoperative consultations with the help of self-reported HRQoL assessments could identify specific domains in which a patient scores poorly, allowing for possible targeted interventions such as prehabilitation programs to improve physical and mental health before surgery. A previous study demonstrates that prehabilitation prior to cardiac surgery significantly improves preoperative physical and mental status and reduces the postoperative complications.^21^ By integrating prehabilitation into the preoperative care pathway, it may be possible to optimize patients' preoperative health status and, consequently, reduce the risk of 30-day postoperative mortality.

### Future perspectives

The primary aim of this study was to explore the possible association between self-reported HRQoL and 30-day mortality. The analyses were intentionally exploratory and hypothesis-generating. To draw more definitive conclusions, future research should aim to validate these results using prospectively and adequately powered data sets to examine individual postoperative complications and their specific contribution to mortality. Such studies should also include time-stamped complication data to clarify the temporal relationship between complication onset and death. Future research should explore the long-term impact of preoperative QoL on survival outcomes, including whether changes in HRQoL trajectories influence survival. Finally, prioritizing HRQoL as a primary outcome in future studies will help confirm the stability and generalizability of these cluster profiles in independent cohorts, and to further encourage exploration of its potential role in risk assessment.

### Strengths and limitations

The strengths of this study include its large sample size, prospective data collection, and the inclusion of a broad spectrum of cardiac surgical procedures and underlying pathologies. Unlike previous studies that primarily focus on specific procedures, such as CABG,^5^^,^^16^^,^^24^ our study includes all cardiac surgery patients who completed the SF-36v2 survey. This allows for generalizable conclusions across the entire cardiac surgery population.

However, several limitations of this study should be acknowledged. First, our conclusions were based on domain-level differences and a data-driven clustering approach rather than exclusively on the established SF-36v2 summary measures. Although this method allowed us to identify distinct and clinically meaningful cluster profiles, it also introduces limitations. Two-step cluster analysis is sensitive to the selection and scaling of input variables, potentially affecting reproducibility and interpretability. However, the large sample size in our study increases the robustness of the results and reduces the influence of small fluctuations in individual variables. Nevertheless, future studies are needed to confirm the stability and generalizability of these cluster profiles in independent cohorts. Second, a CM endpoint was used to explore the relationship between postoperative complications and early mortality, partly due to the low event rate. However, the individual components of this composite outcome are clinically heterogeneous. As a result, it is difficult to determine which specific component contributed most to the observed differences between the 2 clusters. Importantly, the primary aim of this analysis was to explore a potential association between better preoperative HRQoL and postoperative resilience in the context of complications, rather than to identify the contribution of individual complication types. The use of a composite endpoint therefore enabled the evaluation of this hypothesized association, although it precludes definitive conclusions regarding which specific components drive the observed differences between clusters.

Third, the present study focused on a relatively short postoperative follow-up period of 30 days, precluding conclusions regarding the potential impact of preoperative HRQoL on longer-term mortality. Fourth, given that preoperative SF-36v2 assessments are required, patients undergoing emergency procedures were not included, as they do not have the opportunity to complete the questionnaire due to the acute setting. Consequently, our findings cannot be generalized to this patient population. Additionally, our study is conducted at a single tertiary referral center, which may introduce selection bias. However, this risk is limited because of the large sample size, diverse patient population and inclusion of various cardiac procedures, enhancing the generalizability of our findings. Lastly, this study only includes patients who completed the questionnaire. Compared with nonrespondents, participants were slightly older, more often female, had a lower EuroSCORE II, and were more likely to undergo aortic valve surgery. Although these differences were modest, they highlight that our cohort may overrepresent patients with slightly lower surgical risk and certain procedure types, which may limit the generalizability of our findings to all patients undergoing elective cardiac surgery. Those who completed it might be younger and fitter, which introduces a selection bias.

## Conclusions

This study demonstrates a significant association between lower preoperative self-reported health domain scores and higher 30-day mortality in elective cardiac surgery patients. This association appears to be primarily driven by differences in physical health status. Moreover, among patients who developed postoperative complications, mortality was markedly higher in those with lower preoperative HRQoL, suggesting that patients with better preoperative QoL may possess greater mental and physical resilience to recover from postoperative complications. These findings highlight the potential valuable role of quality-of-life assessments, particularly physical health, in preoperative risk stratification and clinical decision-making, beyond conventional surgical risk scores.

Perspectives **COMPETENCY IN MEDICAL KNOWLEDGE:** Preoperative self-reported health status (SF-36v2) provides independent prognostic information about 30-day mortality in elective cardiac surgery patients. By capturing multidimensional factors, including frailty, psychosocial functioning, and physiological reserve, that are only partially represented in established surgical risk scores, HRQoL may serve as a valuable addition to preoperative risk assessment. Integrating HRQoL into routine evaluation may enhance individualized risk stratification and improve the assessment of early postoperative mortality risk.

**TRANSLATIONAL OUTLOOK:** Future research should evaluate whether preoperative HRQoL can provide additional information on long-term postoperative risk beyond the 30-day period and clarify how changes in HRQoL, either deterioration or improvement, affect postoperative outcomes. Multicenter investigations should also assess whether combining HRQoL with established surgical risk scores enhances risk assessment and supports more personalized perioperative care.

## Funding Support and Author Disclosures

Dr Sardari Nia is the CEO of Simurghy (a simulation company); and serves as a consultant for Edwards Lifesciences, Medtronic, Abbott, and Neochord (within the past 36 months). All other authors have reported that they have no relationships relevant to the contents of this paper to disclose.
