# Supplementary material for: Self-Reported Quality of Life and 30-Day Mortality in Elective Cardiac Surgery
Source: JACC Adv. 2026 May 27;5(5):102768. doi: 10.1016/j.jacadv.2026.102768 (PMC13221867; doi:10.1016/j.jacadv.2026.102768)
Supplement: Supplemental Material [file mmc1.docx]

**Supplementary material**

Supplemental methods. Scoring procedure algorithm.

Several items within the SF-36v2 survey require reverse scoring, as they are negatively worded: where higher precoded values reflect poorer health. Reverse coding is a method applied to ensure that higher scores consistently represent better health status on all the items.

Table 1. SF36v2 Response Choices and Scoring Information.

| Reverse scored items | Response Choice | Precoded Response Value | Final Response Value |
| --- | --- | --- | --- |
| 1. In general, would you say your health is: | Excellent | 1 | 5 |
|  | Very good | 2 | 4 |
|  | Good | 3 | 3 |
|  | Fair | 4 | 2 |
|  | Poor | 5 | 1 |
| 2. COMPARED TO ONE YEAR AGO, how would you rate your health in general  NOW? | Much better now than a year ago | 1 | 5 |
|  | Somewhat better now than a year ago | 2 | 4 |
|  | About the same as one year ago | 3 | 3 |
|  | Somewhat worse now than one year ago | 4 | 2 |
|  | Much worse now than one year ago | 5 | 1 |
| 6. during the past 4 weeks, to what extent has your physical health or emotional problems interfered with your normal social activities? | Not at all | 1 | 5 |
|  | Slightly | 2 | 4 |
|  | Moderately | 3 | 3 |
|  | Quite | 4 | 2 |
|  | Extremely | 5 | 1 |
| 7.How much BODILY pain have you had during the PAST 4 WEEKS? | None | 1 | 6 |
|  | Very mild | 2 | 5 |
|  | Mild | 3 | 4 |
|  | Moderate | 4 | 3 |
|  | Severe | 5 | 2 |
|  | Very Severe | 6 | 1 |
| 8.During the PAST 4 WEEKS, how much did PAIN interfere with your normal work  (including both work outside the home and housework)? | Not at all | 1 | 5 |
|  | A little bit | 2 | 4 |
|  | Moderately | 3 | 3 |
|  | Quite a bit | 4 | 2 |
|  | Extremely | 5 | 1 |
| 9a Did you feel full of life? | All of the time | 1 | 5 |
|  | Most of the time | 2 | 4 |
|  | Some of the time | 3 | 3 |
|  | A little of the time | 4 | 2 |
|  | None of the time | 5 | 1 |
| 9h. Have you been happy? | All of the time | 1 | 5 |
|  | Most of the time | 2 | 4 |
|  | Some of the time | 3 | 3 |
|  | A little of the time | 4 | 2 |
|  | None of the time | 5 | 1 |
| 11b.I am as healthy as anybody I know  Mostly true | Definitely true | 1 | 5 |
|  | Mostly true | 2 | 4 |
|  | Don't know | 3 | 3 |
|  | Mostly false | 4 | 2 |
|  | Definitely false | 5 | 1 |
| 11d. My health is excellent | Definitely true | 1 | 5 |
|  | Mostly true | 2 | 4 |
|  | Don't know | 3 | 3 |
|  | Mostly false | 4 | 2 |
|  | Definitely false | 5 | 1 |

**Substituting person-specific estimates for missing items.**

For multi-item scales, the Half-Scale Rule was applied, allowing a scale score to be calculated if at least 50% of the items were completed. Missing values in such cases were imputed based on the respondent’s answers to completed items within the same scale. However, due to the hierarchical structure of items in the Physical Functioning (PF) scale, this approach is not recommended. For the PF scale, item response theory (IRT) methods are preferable when estimating missing values. In this study, cases with incomplete PF item responses were excluded from further analysis.

***Total raw score* for each health domain scale.**

The total raw score is the simple algebraic sum of the final response values for all the items in a given scale after resolving items with missing data. See Table 2.1 *Abbreviated Item Content for the SF-36v2 Health Domain Scales* published in User's manual for the SF-36v2 health survey (3rd ed.) (11).

Responses to this item are treated as ordinal level data that can be used to analyze the percentage of respondents who select each response choice or to estimate the measured change (observed changes in health domain scale scores) reported for each response category.

**Transforming Health Domain Scale Total Raw Scores to 0–100 Scores**

To standardize scale scores, raw totals were transformed to a 0–100 scale using the following formula:

| $Transformed scale score= \frac{(Actual raw score - Lowest possible raw score)}{Possible raw score range}\times100$ | 1 |
| --- | --- |

The SET item was excluded from both raw and transformed score calculations.

**Transforming Health Domain Scale 0–100 Scores to *T* Scores**

The first step is standardizing each SF-36v2 health domain scale using a *z*-score transformation. In User's manual for the SF-36v2 health survey (11) recommended to perform Z-score transformation by subtracting each health domain scale’s 2009 U.S. general population mean from

the 0–100 score for that scale, and then dividing the difference by the given scale’s standard deviation, where HD_i_ ​is the health domain scale’s 0–100 score, and Mean_GP_ and STD_GP_ are the 2009 U.S. general population mean and standard deviation for that domain.

| HD_i_*z* = (HD_i_ - Mean_GP_)/ STD_GP_ | 2 |
| --- | --- |

Further, these standard scores can be put into any convenient form. One possible transformation is T-score transformation (mean = 50, *SD* = 10). To do so, it is necessary to multiply each *z* score by 10, and then add or subtract 50 to/from the resulting product (28). We used an addition of 50 to the resulting product, therefore using the following formula:

| HD_scale_ T score = HD_i_ *z*  × 10 + 50 | 3 |
| --- | --- |

With T scores, each scale has the same average (50) and the same standard deviation (10), meaning each point equals one-tenth of a standard deviation. As a result, without referring to tables of norms, this method makes it clear that health status is within the “average” or “normal” range, whenever group level scores are within 0.3 SD, or 3 T-score points, of the mean.

Using T scores offers a benefit. The use of the *T*-score metric made it possible for the results from the two SF-36 versions to be directly compared, with the mean and standard deviation being 50 and 10, respectively, in the 1998 U.S. general population. However, with time or even in different method surveys, conducting these norms could not be appropriate in all contexts (29).

In this study, given our focus on a specific population, patients undergoing cardiac surgery, we used linearly derived, population-specific standard scores instead of those based on the 1998 or 2009 U.S. general population norms.

**Scoring the Physical and Mental Component Summary Measures.**

The Physical Component Summary (PCS) and Mental Component Summary (MCS) scores were calculated by multiplying each health domain’s z-score by its respective factor scoring coefficient (physical or mental) and summing the resulting products. Orthogonal scoring coefficients were used, as recommended in SF-36v2 development literature (11, 29, 30). These physical and mental component scores were transformed into T-scores using the same method described above.

Supplemental. Table 2. Health domain T-scores of survivors and non-survivors.

|  | Survivors  N= 2028 | | Non-survivors  N= 231 | | P-value |
| --- | --- | --- | --- | --- | --- |
|  | Mean | SD | Mean | SD |  |
| Physical Functioning (PF) | 50.6 | 9.9 | 45.3 | 10.1 | <0.001 |
| Role-Physical (RP) | 50.3 | 10.0 | 47.4 | 9.6 | <0.001 |
| Bodily Pain (BP) | 50.0 | 9.9 | 49.9 | 10.5 | 0.967 |
| General Health (GH) | 50.3 | 9.9 | 47.5 | 10.2 | <0.001 |
| Vitality (VT) | 50.2 | 10.0 | 48.0 | 9.8 | 0.001 |
| Social Functioning (SF) | 50.2 | 9.9 | 48.4 | 10.5 | 0.013 |
| Role-Emotional (RE) | 50.1 | 10.0 | 49.3 | 10.2 | 0.243 |
| Mental Health (MH) | 50.1 | 10.0 | 49.5 | 10.1 | 0.379 |
|  |  |  |  |  |  |
| Physical Component Summary (PCS) | 50.4 | 9.3 | 46.7 | 9.3 | <0.001 |
| Mental Component Summary (MCS) | 50.0 | 9.8 | 50.0 | 10.0 | 0.938 |

Supplemental Figure 1. Physical and mental health status in both clusters compared to the normal range.

| 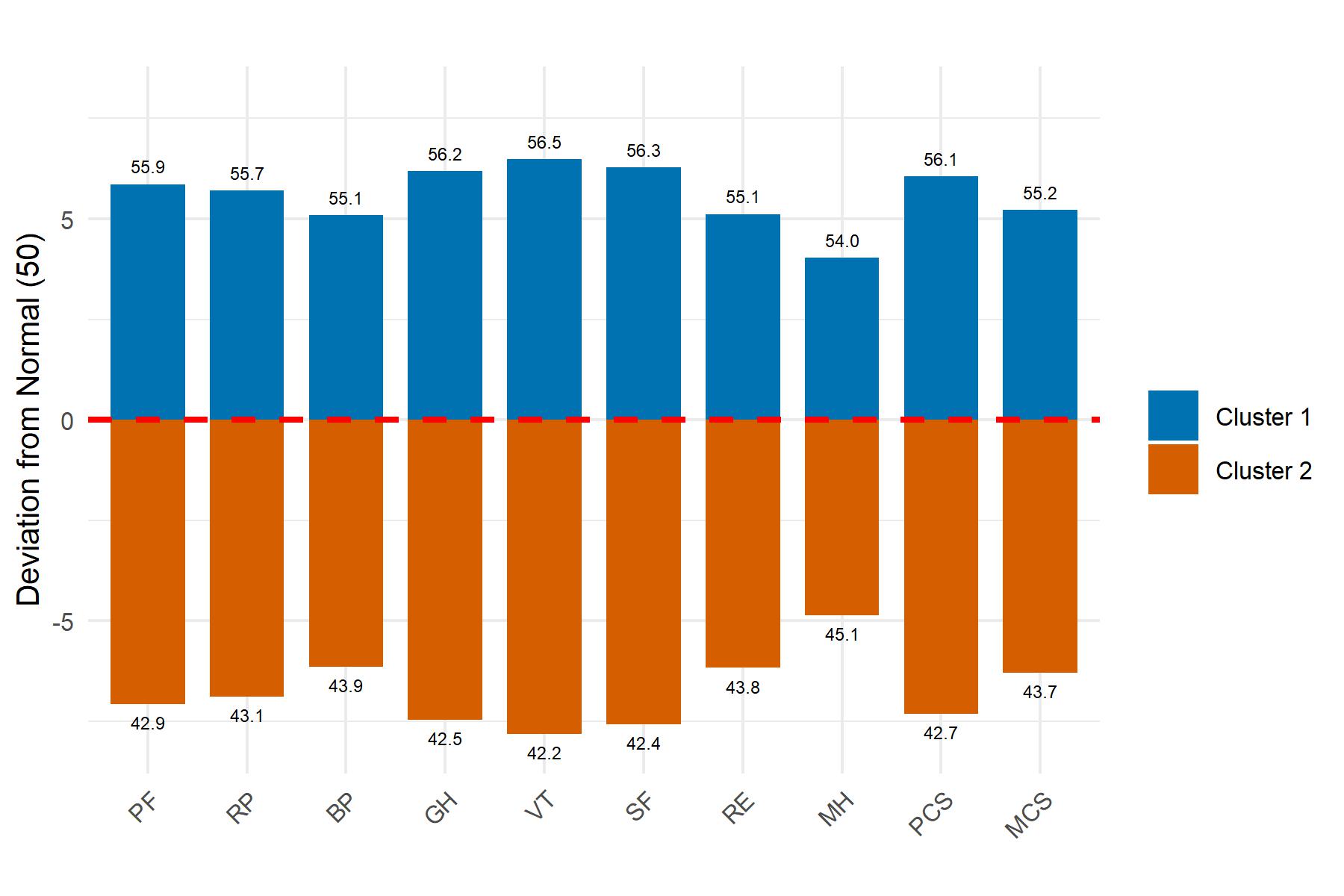 |
| --- |
| \| Figure 1. Physical and mental health status before surgery in Cluster 1 and Cluster 2 compared to the normal range. \| \| --- \| \| The differences between all values in clusters are statistically significant (P < 0.001). All values in clusters are statistically different from "normal value" (one-sample T-test, p < 0.001)  “normal” range  PF Physical Functioning  RP Role-Physical  BP Bodily Pain  GH General Health  VT Vitality  SF Social Functioning  RE Role-Emotional  MH Mental Health  PCS Physical Component Summary  MCS Mental Component Summary \| |
